# Dynamic Opial diamond-*α* integral inequalities involving the power of a function

**DOI:** 10.1186/s13660-017-1411-2

**Published:** 2017-06-17

**Authors:** Tatjana Z Mirković

**Affiliations:** College of Applied Professional Studies, Filipa Filipovića 20, Vranje, 17000 Serbia

**Keywords:** 34N05, 26D10, Opial-type inequality, time scale

## Abstract

In this paper, we present some new dynamic Opial-type diamond alpha inequalities on time scales. The obtained results are related to the function $f^{k}$.

## Introduction

A time scale $\mathbb{T}$ is an arbitrary nonempty closed subset of real numbers. For $t \in\mathbb{T}$, we define the forward jump operator $\sigma:\mathbb{T} \to\mathbb{T}$ by $\sigma ( t ) := \inf \{ s \in\mathbb{T}:s > t \} $ and the backward jump operator $\rho:\mathbb{T} \to\mathbb{T}$ by $\rho ( t ) := \sup \{ s \in\mathbb{T}:s < t \} $. If $\sigma ( t ) > t$, we say that *t* is right-scattered, whereas if $\rho ( t ) < t$, we say that *t* is left-scattered. Points that are simultaneously right-scattered and left-scattered are said to be isolated. If $\sigma ( t ) = t$, then *t* is called right-dense; if $\rho ( t ) = t$, then *t* is called left-dense. Points that are right-dense and left-dense at the same time are called dense. The mappings $\mu,\nu:\mathbb{T} \to[ 0,\infty )$, defined by $\mu ( t ) := \sigma ( t ) - t$ and $\nu ( t ) := t - \rho ( t )$, are called the forward and backward graininess function, respectively. If $\mathbb{T}$ has a left-scattered maximum $t_{1} $, then $\mathbb{T}^{k} = \mathbb{T} - \{ t_{1} \} $, otherwise $\mathbb{T}^{k} = \mathbb{T}$. If $\mathbb{T}$ has a right-scattered minimum $t_{2} $, then $\mathbb{T}_{k} = \mathbb{T} - \{ t_{2} \} $, otherwise $\mathbb{T}_{k} = \mathbb{T}$. Finally, $\mathbb{T}_{k}^{k} = \mathbb{T}^{k} \cap\mathbb{T}_{k} $.

### Theorem 1.1

*Assume* $f,g:\mathbb{T} \to\mathbb{R}$ *are delta differentiable at* $t \in\mathbb{T}^{k}$. *Then*:

1. *The sum* $f + g:\mathbb{T} \to\mathbb{R}$ *is delta differentiable at* *t* *with*
  $$( f + g )^{\Delta}( t ) = f^{\Delta}( t ) + g^{\Delta}( t ). $$
2. *For any constant* *α*, $\alpha f:\mathbb{T} \to \mathbb{R}$ *is delta differentiable at* *t* *with*
  $$( \alpha f )^{\Delta}( t ) = \alpha f^{\Delta}( t ). $$
3. *The product* $fg:\mathbb{T} \to\mathbb{R}$ *is delta differentiable at* *t* *with*
  $$( fg )^{\Delta}( t ) = f^{\Delta}( t )g ( t ) + f^{\sigma}( t )g^{\Delta}( t ) = f ( t )g^{\Delta}( t ) + f^{\Delta}( t )g^{\sigma}( t ). $$

### Theorem 1.2

*Assume* $f,g:\mathbb{T} \to\mathbb{R}$ *are nabla differentiable at* $t \in\mathbb{T}_{k}$. *Then*:

1. *The sum* $f + g:\mathbb{T} \to\mathbb{R}$ *is nabla differentiable at* *t* *with*
  $$( f + g )^{\nabla}( t ) = f^{\nabla}( t ) + g^{\nabla}( t ). $$
2. *For any constant* *α*, $\alpha f:\mathbb{T} \to\mathbb{R}$ *is nabla differentiable at* *t* *with*
  $$( \alpha f )^{\nabla}( t ) = \alpha f^{\nabla}( t ). $$
3. *The product* $fg:\mathbb{T} \to \mathbb{R}$ *is nabla differentiable at* *t* *with*
  $$( fg )^{\nabla}( t ) = f^{\nabla}( t )g ( t ) + f^{\rho}( t )g^{\nabla}( t ) = f ( t )g^{\nabla}( t ) + f^{\nabla}( t )g^{\rho}( t ). $$

The following formulas will be used in our paper:

$$\begin{aligned}& \bigl( f^{l + 1} \bigr)^{\Delta}= \Biggl\{ \sum _{k = 0}^{l} f^{k} \bigl( f^{\sigma}\bigr)^{l - k} \Biggr\} f^{\Delta}, \quad l \in N, \\ & \bigl( f^{l + 1} \bigr)^{\nabla}= \Biggl\{ \sum _{k = 0}^{l} f^{k} \bigl( f^{\rho}\bigr)^{l - k} \Biggr\} f^{\nabla}, \quad l \in N. \end{aligned}$$

### Definition 1.3

Let $0 \le\alpha \le1$ and let *f* be both delta and nabla differentiable at $t \in\mathbb{T}_{k}^{k}$. Then *f* is diamond-*α* differentiable at *t* and $f^{\diamondsuit _{\alpha}} ( t ) = \alpha f^{\Delta}( t ) + ( 1 - \alpha )f^{\nabla}( t )$.

### Definition 1.4

Let $a,b \in\mathbb{T}$, $a < b$, $f:\mathbb{T} \to\mathbb{R}$ and $\alpha \in [ 0,1 ]$. The diamond-*α* integral of *t* on $[ a,b ]_{\mathbb{T}}$ is defined by

$$\int _{a}^{b} f ( t )\diamondsuit_{\alpha}t = \alpha \int _{a}^{b} f ( t )\Delta t + ( 1 - \alpha ) \int _{a}^{b} f ( t )\nabla t. $$

### Theorem 1.5

*Let* $f,g:\mathbb{T} \to\mathbb{R}$ *be* $\diamondsuit _{\alpha}$-*differentiable at* $t \in\mathbb{T}$. *Then*

1. $f + g$ *is* $\diamondsuit_{\alpha}$-*differentiable* $t \in \mathbb{T}$ *with* $( f + g )^{\diamondsuit_{\alpha}} = f^{\diamondsuit_{\alpha}} + g^{\diamondsuit_{\alpha}}$,
2. *fg* *is* $\diamondsuit_{\alpha}$-*differentiable at* $t \in \mathbb{T}$ *with* $( fg )^{\diamondsuit_{\alpha}} = f^{\diamondsuit_{\alpha}} g + \alpha f^{\sigma}g^{\Delta}+ ( 1 - \alpha )f^{\rho}g^{\nabla}$.

Many authors have studied the theory of integral inequalities on time scales (see, for example, [1–10]). In [3], the following Opial inequality on time scales was established.

### Theorem 1.6

[3]

*For a delta differentiable* $f: [ 0,h ] \cap\mathbb{T} \to\mathbb{R}$ *with* $f ( 0 ) = 0$, *we have*

1 $$ \int _{0}^{h} \bigl\vert { \bigl( f + f^{\sigma}\bigr)f^{\Delta}} \bigr\vert \Delta t \le h \int _{0}^{h} \bigl\vert {f^{\Delta}} \bigr\vert ^{2} \Delta t, $$

*with equality when* $f ( t ) = ct$.

In [1], the authors established the following theorem.

### Theorem 1.7

[1]

*Let* $\omega ( t )$ *be positive and continuous on* $( 0,h )$ *with* $\int _{0}^{h} \omega^{1 - q} \Delta t < \infty$, $q > 1$. *For a differentiable* $f: [ 0,h ] \to\mathbb{R}$ *with* $f ( 0 ) = 0$, *we have*

$$\int _{0}^{h} \bigl\vert \bigl( f + f^{\sigma}\bigr)f^{\Delta}\bigr\vert \Delta t \le \biggl( \int _{0}^{h} \omega^{1 - q} \Delta t \biggr)^{\frac{2}{q}} \biggl( \int _{0}^{h} \omega \bigl\vert f^{\Delta}\bigr\vert ^{p} \Delta t \biggr)^{\frac{2}{p}}, $$

*where* $p > 1$ *and* $\frac{1}{p} + \frac{1}{q} = 1$, *and with equality when* $f ( t ) = c\int_{0}^{t} \omega^{1 - q} \Delta\tau$ *for a constant c*.

## Main results

In this section, we present our results.

### Theorem 2.1

*Let* *T* *be a time scale*. *For* $\diamondsuit_{\alpha}$ *differentiable* $f: [ 0,h ] \cap T \to R$, *with* $f ( 0 ) = 0$ *we have*

2 $$ \int _{0}^{h} \bigl\vert {f^{k} } \bigr\vert ^{\diamondsuit_{\alpha}} ( t )\diamondsuit_{\alpha}t \le h^{k - 1} \int _{0}^{h} \bigl\vert {f^{\diamondsuit_{\alpha}} } \bigr\vert ^{k} ( t )\diamondsuit_{\alpha}t. $$

### Proof

Starting with the left side of (2), we obtain

$$\begin{aligned} \int _{0}^{h} \bigl\vert f^{k} \bigr\vert ^{\diamondsuit_{\alpha}} ( t )\diamondsuit _{\alpha}( t ) =& \int _{0}^{h} \bigl\vert f \cdot f^{k - 1} \bigr\vert ^{\diamondsuit_{\alpha}} ( t )\diamondsuit_{\alpha}( t ) \\ =& \int _{0}^{h} \bigl\vert f^{k - 1} f^{\diamondsuit_{\alpha}} + \alpha f^{\sigma}\bigl( f^{k - 1} \bigr)^{\Delta}+ ( 1 - \alpha )f^{\rho}\bigl( f^{k - 1} \bigr)^{\nabla}\bigr\vert ( t )\diamondsuit_{\alpha}( t ) \\ =& \alpha \int _{0}^{h} \bigl\vert f^{k - 1} f^{\diamondsuit _{\alpha}} + \alpha f^{\sigma}\bigl( f^{k - 1} \bigr)^{\Delta}+ ( 1 - \alpha )f^{\rho}\bigl( f^{k - 1} \bigr)^{\nabla}\bigr\vert ( t )\Delta t \\ &{}+ ( 1 - \alpha ) \int _{0}^{h} \bigl\vert f^{k - 1} f^{\diamondsuit_{\alpha}} + \alpha f^{\sigma}\bigl( f^{k - 1} \bigr)^{\Delta}+ ( 1 - \alpha )f^{\rho}\bigl( f^{k - 1} \bigr)^{\nabla}\bigr\vert ( t )\nabla t \\ \le&\alpha \int _{0}^{h} \bigl\vert f^{k - 1} f^{\diamondsuit _{\alpha}} \bigr\vert ( t )\Delta t + \alpha^{2} \int _{0}^{h} \bigl\vert f^{\sigma}\bigl( f^{k - 1} \bigr)^{\Delta}\bigr\vert ( t )\Delta t \\ & {}+ \alpha ( 1 - \alpha ) \int _{0}^{h} \bigl\vert f^{\rho}\bigl( f^{k - 1} \bigr)^{\nabla}\bigr\vert ( t )\Delta t + ( 1 - \alpha ) \int _{0}^{h} \bigl\vert f^{k - 1} f^{\diamondsuit_{\alpha}} \bigr\vert ( t )\nabla t \\ & {}+ \alpha ( 1 - \alpha ) \int _{0}^{h} \bigl\vert f^{\sigma}\bigl( f^{k - 1} \bigr)^{\Delta}\bigr\vert ( t )\nabla t + ( 1 - \alpha )^{2} \int _{0}^{h} \bigl\vert f^{\rho}\bigl( f^{k - 1} \bigr)^{\nabla}\bigr\vert ( t )\nabla t. \end{aligned}$$

Using Definition 1.3, we get

$$\begin{aligned} \int _{0}^{h} \bigl\vert f^{k} \bigr\vert ^{\diamondsuit_{\alpha}} ( t )\diamondsuit _{\alpha}( t ) \le&\alpha \int _{0}^{h} \bigl\vert \alpha f^{k - 1} f^{\Delta}+ ( 1 - \alpha )f^{k - 1} f^{\nabla}\bigr\vert ( t )\Delta t \\ & {}+ \alpha^{2} \int _{0}^{h} \bigl\vert f^{\sigma}\bigl( f^{k - 1} \bigr)^{\Delta}\bigr\vert ( t )\Delta t + \alpha ( 1 - \alpha ) \int _{0}^{h} \bigl\vert f^{\rho}\bigl( f^{k - 1} \bigr)^{\nabla}\bigr\vert ( t )\Delta t \\ & {}+ ( 1 - \alpha ) \int _{0}^{h} \bigl\vert \alpha f^{k - 1} f^{\Delta}+ ( 1 - \alpha )f^{k - 1} f^{\nabla}\bigr\vert ( t )\nabla t \\ & {}+ \alpha ( 1 - \alpha ) \int _{0}^{h} \bigl\vert f^{\sigma}\bigl( f^{k - 1} \bigr)^{\Delta}\bigr\vert ( t )\nabla t + ( 1 - \alpha )^{2} \int _{0}^{h} \bigl\vert f^{\rho}\bigl( f^{k - 1} \bigr)^{\nabla}\bigr\vert ( t )\nabla t \\ \le&\alpha^{2} \int _{0}^{h} \bigl\vert f^{k - 1} f^{\Delta}\bigr\vert ( t )\Delta t + \alpha ( 1 - \alpha ) \int _{0}^{h} \bigl\vert f^{k - 1} f^{\nabla}\bigr\vert ( t )\Delta t \\ & {}+ \alpha^{2} \int _{0}^{h} \bigl\vert f^{\sigma}\bigl( f^{k - 1} \bigr)^{\Delta}\bigr\vert ( t )\Delta t + \alpha ( 1 - \alpha ) \int _{0}^{h} \bigl\vert f^{\rho}\bigl( f^{k - 1} \bigr)^{\nabla}\bigr\vert ( t )\Delta t \\ & {}+ \alpha ( 1 - \alpha ) \int _{0}^{h} \bigl\vert f^{k - 1} f^{\Delta}\bigr\vert ( t )\nabla t + ( 1 - \alpha )^{2} \int _{0}^{h} \bigl\vert f^{k - 1} f^{\nabla}\bigr\vert ( t )\nabla t \\ & {}+ \alpha ( 1 - \alpha ) \int _{0}^{h} \bigl\vert f^{\sigma}\bigl( f^{k - 1} \bigr)^{\Delta}\bigr\vert ( t )\nabla t + ( 1 - \alpha )^{2} \int _{0}^{h} \bigl\vert f^{\rho}\bigl( f^{k - 1} \bigr)^{\nabla}\bigr\vert ( t )\nabla t. \end{aligned}$$

We find that

$$\begin{aligned} \int _{0}^{h} \bigl\vert f^{\sigma}\bigl( f^{k - 1} \bigr)^{\Delta}\bigr\vert ( t )\Delta t =& \int _{0}^{h} \bigl\vert f^{\sigma}\bigl( f \cdot f^{k - 2} \bigr)^{\Delta}\bigr\vert ( t ) \Delta t \\ =& \int _{0}^{h} \bigl\vert f^{\sigma}\bigl( f^{\Delta}f^{k - 2} + f^{\sigma}\bigl( f \cdot f^{k - 3} \bigr)^{\Delta}\bigr) \bigr\vert ( t )\Delta t \\ & {}\vdots \\ =& \int _{0}^{h} \bigl\vert f^{\sigma}\bigl( f^{\Delta}f^{k - 2} + f^{\sigma}f^{\Delta}f^{k - 3} + \cdots+ \bigl( f^{\sigma}\bigr)^{2} f^{\Delta}\bigr) \bigr\vert ( t )\Delta t \\ =& \int _{0}^{h} \bigl\vert f^{\sigma}f^{k - 2} + \bigl( f^{\sigma}\bigr)^{2} f^{k - 3} + \cdots+ \bigl( f^{\sigma}\bigr) \bigr\vert \bigl\vert f^{\Delta}\bigr\vert ( t )\Delta t \\ =& \int _{0}^{h} \Biggl\vert \sum _{n = 0}^{k - 2} f^{n} \bigl( f^{\sigma}\bigr)^{k - 1 - n} \Biggr\vert \bigl\vert f^{\Delta}\bigr\vert ( t )\Delta t. \end{aligned}$$

Similarly,

$$\int _{0}^{h} \bigl\vert f^{\rho}\bigl( f^{k - 1} \bigr)^{\nabla}\bigr\vert ( t )\Delta t = \int _{0}^{h} \Biggl\vert \sum _{n = 0}^{k - 2} f^{n} \bigl( f^{\rho}\bigr)^{k - 1 - n} \Biggr\vert \bigl\vert f^{\nabla}\bigr\vert ( t )\nabla t. $$

Therefore,

$$\begin{aligned} \int _{0}^{h} \bigl\vert f^{k} \bigr\vert ^{\diamondsuit _{\alpha}} ( t )\diamondsuit_{\alpha}t \le& \alpha^{2} \int _{0}^{h} \bigl\vert f^{k - 1} f^{\Delta}\bigr\vert ( t )\Delta t + \alpha ( 1 - \alpha ) \int _{0}^{h} \bigl\vert f^{k - 1} f^{\nabla}\bigr\vert ( t )\Delta t \\ & {}+ \alpha^{2} \int _{0}^{h} \Biggl\vert \sum _{n = 0}^{k - 2} f^{n} \bigl( f^{\sigma}\bigr)^{k - 1 - n} \Biggr\vert \bigl\vert f^{\Delta}\bigr\vert ( t )\Delta t \\ & {}+ \alpha ( 1 - \alpha ) \int _{0}^{h} \Biggl\vert \sum _{n = 0}^{k - 2} f^{n} \bigl( f^{\rho}\bigr)^{k - 1 - n} \Biggr\vert \bigl\vert f^{\nabla}\bigr\vert ( t )\Delta t \\ & {}+ \alpha ( 1 - \alpha ) \int _{0}^{h} \bigl\vert f^{k - 1} f^{\Delta}\bigr\vert ( t )\nabla t + ( 1 - \alpha )^{2} \int _{0}^{h} \bigl\vert f^{k - 1} f^{\nabla}\bigr\vert ( t )\nabla t \\ & {}+ \alpha ( 1 - \alpha ) \int _{0}^{h} \Biggl\vert \sum _{n = 0}^{k - 2} f^{n} \bigl( f^{\sigma}\bigr)^{k - 1 - n} \Biggr\vert \bigl\vert f^{\Delta}\bigr\vert \nabla t \\ & {}+ ( 1 - \alpha )^{2} \int _{0}^{h} \Biggl\vert \sum _{n = 0}^{k - 2} f^{n} \bigl( f^{\rho}\bigr)^{k - 1 - n} \Biggr\vert \bigl\vert f^{\nabla}\bigr\vert ( t )\nabla t \\ = &\alpha^{2} \int _{0}^{h} \Biggl( \bigl\vert f^{k - 1} \bigr\vert + \Biggl\vert \sum_{n = 0}^{k - 2} f^{n} \bigl( f^{\sigma}\bigr)^{k - 1 - n} \Biggr\vert \Biggr) \bigl\vert f^{\Delta}( t ) \bigr\vert \Delta t \\ & {}+ \alpha ( 1 - \alpha ) \int _{0}^{h} \Biggl( \bigl\vert f^{k - 1} \bigr\vert + \Biggl\vert \sum_{n = 0}^{k - 2} f^{n} \bigl( f^{\rho}\bigr)^{k - 1 - n} \Biggr\vert \Biggr) \bigl\vert f^{\nabla}( t ) \bigr\vert \Delta t \\ & {}+ \alpha ( 1 - \alpha ) \int _{0}^{h} \Biggl( \bigl\vert f^{k - 1} \bigr\vert + \Biggl\vert \sum_{n = 0}^{k - 2} f^{n} \bigl( f^{\sigma}\bigr)^{k - 1 - n} \Biggr\vert \Biggr) \bigl\vert f^{\Delta}( t ) \bigr\vert \nabla t \\ & {}+ ( 1 - \alpha )^{2} \int _{0}^{h} \Biggl( \bigl\vert f^{k - 1} \bigr\vert + \Biggl\vert \sum_{n = 0}^{k - 2} f^{n} \bigl( f^{\rho}\bigr)^{k - 1 - n} \Biggr\vert \Biggr) \bigl\vert f^{\nabla}( t ) \bigr\vert \nabla t \\ \le&\alpha^{2} \int _{0}^{h} \Biggl( \bigl\vert f^{k - 1} \bigr\vert + \sum_{n = 0}^{k - 2} \bigl\vert f^{n} \bigl( f^{\sigma}\bigr)^{k - 1 - n} \bigr\vert \Biggr) \bigl\vert f^{\Delta}( t ) \bigr\vert \Delta t \\ & {}+ \alpha ( 1 - \alpha ) \int _{0}^{h} \Biggl( \bigl\vert f^{k - 1} \bigr\vert + \sum_{n = 0}^{k - 2} \bigl\vert f^{n} \bigl( f^{\rho}\bigr)^{k - 1 - n} \bigr\vert \Biggr) \bigl\vert f^{\nabla}( t ) \bigr\vert \Delta t \\ & {}+ \alpha ( 1 - \alpha ) \int _{0}^{h} \Biggl( \bigl\vert f^{k - 1} \bigr\vert + \sum_{n = 0}^{k - 2} \bigl\vert f^{n} \bigl( f^{\sigma}\bigr)^{k - 1 - n} \bigr\vert \Biggr) \bigl\vert f^{\Delta}( t ) \bigr\vert \nabla t \\ & {}+ ( 1 - \alpha )^{2} \int _{0}^{h} \Biggl( \bigl\vert f^{k - 1} \bigr\vert + \sum_{n = 0}^{k - 2} \bigl\vert f^{n} \bigl( f^{\rho}\bigr)^{k - 1 - n} \bigr\vert \Biggr) \bigl\vert f^{\nabla}( t ) \bigr\vert \nabla t \\ = &\alpha^{2} \int _{0}^{h} \Biggl( \sum _{n = 0}^{k - 1} \bigl\vert f^{n} \bigr\vert \bigl\vert \bigl( f^{\sigma}\bigr)^{k - 1 - n} \bigr\vert \Biggr) \bigl\vert f^{\Delta}( t ) \bigr\vert \Delta t \\ & {}+ \alpha ( 1 - \alpha ) \int _{0}^{h} \Biggl( \sum _{n = 0}^{k - 1} \bigl\vert f^{n} \bigr\vert \bigl\vert \bigl( f^{\rho}\bigr)^{k - 1 - n} \bigr\vert \Biggr) \bigl\vert f^{\nabla}( t ) \bigr\vert \Delta t \\ & {}+ \alpha ( 1 - \alpha ) \int _{0}^{h} \Biggl( \sum _{n = 0}^{k - 1} \bigl\vert f^{n} \bigr\vert \bigl\vert \bigl( f^{\sigma}\bigr)^{k - 1 - n} \bigr\vert \Biggr) \bigl\vert f^{\Delta}( t ) \bigr\vert \nabla t \\ & {}+ ( 1 - \alpha )^{2} \int _{0}^{h} \Biggl( \sum _{n = 0}^{k - 1} \bigl\vert f^{n} \bigr\vert \bigl\vert \bigl( f^{\rho}\bigr)^{k - 1 - n} \bigr\vert \Biggr) \bigl\vert f^{\nabla}( t ) \bigr\vert \nabla t. \end{aligned}$$

Consider $g ( t ) = \int _{0}^{t} \vert f^{\diamondsuit_{\alpha}} ( s ) \vert \diamondsuit _{\alpha}s$. Then we have $g^{\Delta}( t ) = \vert f^{\Delta}( t ) \vert $, $g^{\nabla}( t ) = \vert f^{\nabla}( t ) \vert $, and $\vert f \vert \le g$, so that $g ( t ) = \int _{0}^{t} \vert f^{\diamondsuit_{\alpha}} ( s ) \vert \diamondsuit_{\alpha}s \ge \vert \int _{0}^{t} f^{\diamondsuit_{\alpha}} ( s )\diamondsuit_{\alpha}s \vert = \vert f ( t ) - f ( 0 ) \vert = \vert f ( t ) \vert $.

The above inequality becomes

$$\begin{aligned} \int _{0}^{h} \bigl\vert f^{k} \bigr\vert ^{\diamondsuit_{\alpha}} ( t )\diamondsuit _{\alpha} \le& \alpha^{2} \int _{0}^{h} \Biggl( \sum _{n = 0}^{k - 1} g^{n} \bigl( g^{\sigma}\bigr)^{k - 1 - n} \Biggr) \bigl( g^{\Delta}\bigr) ( t )\Delta t \\ & {}+ \alpha ( 1 - \alpha ) \int _{0}^{h} \Biggl( \sum _{n = 0}^{k - 1} g^{n} \bigl( g^{\rho}\bigr)^{k - 1 - n} \Biggr) \bigl( g^{\nabla}\bigr) ( t )\Delta t \\ & {}+ \alpha ( 1 - \alpha ) \int _{0}^{h} \Biggl( \sum _{n = 0}^{k - 1} g^{n} \bigl( g^{\sigma}\bigr)^{k - 1 - n} \Biggr) \bigl( g^{\Delta}\bigr) ( t )\nabla t \\ & {}+ ( 1 - \alpha )^{2} \int _{0}^{h} \Biggl( \sum _{n = 0}^{k - 1} g^{n} \bigl( g^{\rho}\bigr)^{k - 1 - n} \Biggr) \bigl( g^{\nabla}\bigr) ( t )\nabla t \\ = &\alpha^{2} \int _{0}^{h} \bigl( g^{k} \bigr)^{\Delta}( t )\Delta t + \alpha ( 1 - \alpha ) \int _{0}^{h} \bigl( g^{k} \bigr)^{\nabla}( t )\Delta t \\ & {}+ \alpha ( 1 - \alpha ) \int _{0}^{h} \bigl( g^{k} \bigr)^{\Delta}( t )\nabla t + ( 1 - \alpha )^{2} \int _{0}^{h} \bigl( g^{k} \bigr)^{\nabla}( t )\nabla t \\ = &\alpha \biggl[ \alpha \int _{0}^{h} \bigl( g^{k} \bigr)^{\Delta}\Delta t + ( 1 - \alpha ) \int _{0}^{h} \bigl( g^{k} \bigr)^{\Delta}\nabla t \biggr] \\ & {}+ ( 1 - \alpha ) \biggl[ \int _{0}^{h} \alpha \bigl( g^{k} \bigr)^{\nabla}\Delta t + ( 1 - \alpha ) \int _{0}^{h} \bigl( g^{k} \bigr)^{\nabla}\nabla t \biggr] \\ = &\alpha \int _{0}^{h} \bigl( g^{k} \bigr)^{\Delta}\diamondsuit_{\alpha}+ ( 1 - \alpha ) \int _{0}^{h} \bigl( g^{k} \bigr)^{\nabla}\diamondsuit _{\alpha}= \int _{0}^{h} \bigl( g^{k} \bigr) ( t )^{\diamondsuit_{\alpha}} \diamondsuit_{\alpha}\\ =& g^{k} ( t ) | _{0}^{h} = g^{k} ( h ) - g^{k} ( 0 ) = \bigl[ g ( h ) \bigr]^{k} = \biggl[ \int _{0}^{h} \bigl\vert f^{\diamondsuit _{\alpha}} ( s ) \bigr\vert \diamondsuit_{\alpha}s \biggr]^{k}. \end{aligned}$$

By using Hölder’s inequality with indices $p = \frac{k}{k - 1}$ and $q = k$, we obtain

$$\begin{aligned} \biggl[ \int _{0}^{h} 1 \cdot \bigl\vert {f^{\diamondsuit_{\alpha}} ( s )} \bigr\vert \diamondsuit_{\alpha}s \biggr]^{k} \le& \biggl[ \biggl( \int _{0}^{h} 1^{\frac{k}{k - 1}} \diamondsuit _{\alpha}s \biggr)^{\frac{k - 1}{k}} \biggl( \int _{0}^{h} \bigl\vert {f^{\diamondsuit_{\alpha}} ( s )} \bigr\vert ^{k} \diamondsuit_{\alpha}s \biggr)^{\frac{1}{k}} \biggr]^{k} \\ = & \biggl( \int _{0}^{h} \diamondsuit_{\alpha}s \biggr)^{k - 1} \biggl( \int _{0}^{h} \bigl\vert {f^{\diamondsuit _{\alpha}} ( s )} \bigr\vert ^{k} \diamondsuit_{\alpha}s \biggr) \\ = & \bigl( s |_{0}^{h} \bigr)^{k - 1} \int _{0}^{h} \bigl\vert {f^{\diamondsuit_{\alpha}} ( s )} \bigr\vert ^{k} \diamondsuit_{\alpha}s \\ = &h^{k - 1} \int _{0}^{h} \bigl\vert {f^{\diamondsuit_{\alpha}} ( s )} \bigr\vert ^{k} \diamondsuit_{\alpha}s, \end{aligned}$$

hence the proof is complete. □

### Theorem 2.2

*Let* $\omega ( t )$ *be positive and continuous on* $( 0,h )$, *with* $\int _{0}^{h} \omega^{1 - q} ( t )\Delta t < \infty$, $q > 1$. *For differentiable* $f: [ 0,h ] \to\mathbb{R}$ *with* $f ( 0 ) = 0$ *we have*

3 $$ \int _{0}^{h} \bigl\vert {f^{k} } \bigr\vert ^{\Delta}\Delta t \le \biggl( \int _{0}^{h} \omega^{1 - q} \Delta t \biggr)^{\frac{k}{q}} \biggl( \int _{0}^{h} \omega \bigl\vert {f^{\Delta}} \bigr\vert ^{p} \Delta t \biggr)^{\frac{k}{q}}, $$

*where* $p > 1$ *and* $\frac{1}{p} + \frac{1}{q} = 1$.

### Proof

We take $g ( t ) = \int _{0}^{t} \vert f^{\Delta}( s ) \vert \Delta s$. Then $\vert f ( t ) \vert \le g ( t )$, $g^{\Delta}( t ) = \vert f^{\Delta}( t ) \vert $, so we have

$$\begin{aligned} \int _{0}^{h} \bigl\vert {f^{k} } \bigr\vert ^{\Delta}\Delta t =& \int _{0}^{h} \Biggl\vert {\sum _{k = 0}^{n - 1} f^{k} \bigl( f^{\sigma}\bigr)^{n - 1 - k} } \Biggr\vert \bigl\vert {f^{\Delta}} \bigr\vert ( t )\Delta t \\ \le& \int _{0}^{h} \Biggl( \sum _{k = 0}^{n - 1} g^{k} \bigl( g^{\sigma}\bigr)^{n - 1 - k} \Biggr) \bigl( g^{\Delta}\bigr) ( t )\Delta t = \int _{0}^{h} \bigl( g^{k} \bigr)^{\Delta}\Delta t \\ =& g^{k} ( h ) - g^{k} ( 0 ) = g^{k} ( h ) = \biggl( \int _{0}^{h} \bigl\vert {f^{\Delta}} \bigr\vert ( t )\Delta t \biggr)^{k} \\ =& \biggl( \int _{0}^{h} \omega^{ - \frac{1}{p}} \omega ^{\frac{1}{p}} \bigl\vert {f^{\Delta}} \bigr\vert ( t )\Delta t \biggr)^{k} \\ \le& \biggl[ \biggl( \int _{0}^{h} \bigl( \omega^{ - \frac{1}{p}} \bigr)^{q} \Delta t \biggr)^{\frac{1}{q}} \biggl( \int _{0}^{h} \bigl( \omega^{\frac{1}{p}} \bigl\vert {f^{\Delta}} \bigr\vert \bigr)^{p} ( t )\Delta t \biggr)^{\frac{1}{p}} \biggr]^{k} \\ =& \biggl( \int _{0}^{h} \omega^{1 - q} \Delta t \biggr)^{\frac{k}{q}} \biggl( \int _{0}^{h} \bigl( \omega \bigl\vert f^{\Delta}\bigr\vert \bigr)^{p} ( t )\Delta t \biggr)^{\frac{k}{p}}. \end{aligned}$$

 □

### Theorem 2.3

*Let* $\omega ( t )$ *be positive and continuous on* $( 0,h )$, *with* $\int _{0}^{h} \omega^{1 - q} ( t )\nabla t < \infty$, $q > 1$. *For differentiable* $f: [ 0,h ] \to\mathbb{R}$ *with* $f ( 0 ) = 0$ *we have*

4 $$ \int _{0}^{h} \bigl\vert {f^{k} } \bigr\vert ^{\nabla}\nabla t \le \biggl( \int _{0}^{h} \omega^{1 - q} \nabla t \biggr)^{\frac{k}{q}} \biggl( \int _{0}^{h} \omega \bigl\vert {f^{\nabla}} \bigr\vert ^{p} \nabla t \biggr)^{\frac{k}{q}}, $$

*where* $p > 1$ *and* $\frac{1}{p} + \frac{1}{q} = 1$.

### Theorem 2.4

*Assume that* $p > 1$, $q = \frac{p}{p - 1}$, $\alpha \in [ 0,1 ]$, $h \in ( 0,\infty )_{\mathbb{T}}$, $\omega \in\mathbb{C} ( [ 0,h ]_{\mathbb{T}} , ( 0,\infty ) )$ *and* $f \in \mathbb{C}_{\diamondsuit_{\alpha}}^{1} ( [ 0,h ]_{\mathbb{T}} ,\mathbb{R} )$. *If* $\alpha f^{\Delta}\ge 0$, $( 1 - \alpha )f^{\nabla}\ge0$ *and* $f ( 0 ) = 0$ *then*

5 $$\begin{aligned}& \alpha^{k} \int _{0}^{h} \bigl\vert { \bigl( f^{k} \bigr)^{\Delta}( t )} \bigr\vert \Delta t + ( 1 - \alpha )^{k} \int _{0}^{h} \bigl\vert { \bigl( f^{k} \bigr)^{\nabla}( t )} \bigr\vert \nabla t \\& \quad \le \biggl( \int _{0}^{h} \omega^{1 - q} ( t ) \diamondsuit _{\alpha}t \biggr)^{\frac{k}{q}} \biggl( \int _{0}^{h} \omega( t ) \bigl\vert f^{\diamondsuit _{\alpha}} ( t ) \bigr\vert ^{p} \diamondsuit_{\alpha}t \biggr)^{\frac{k}{p}}. \end{aligned}$$

### Proof

By Theorems 2.2, 2.3, Hölder’s inequality and $k = \frac{k}{q} + ( 1 + p )\frac{k}{p}$, we get

$$\begin{aligned}& \alpha^{k} \int _{0}^{h} \bigl\vert \bigl( f^{k} \bigr)^{\Delta}( t ) \bigr\vert \Delta t + ( 1 - \alpha )^{k} \int _{0}^{h} \bigl\vert \bigl( f^{k} \bigr)^{\nabla}( t ) \bigr\vert \nabla t \\& \quad = \alpha^{\frac{k}{q} + ( 1 + p )\frac{k}{p}} \int _{0}^{h} \bigl\vert \bigl( f^{k} \bigr)^{\Delta}( t ) \bigr\vert \Delta t + ( 1 - \alpha )^{\frac{k}{q} + ( 1 + p )\frac{k}{p}} \int _{0}^{h} \bigl\vert \bigl( f^{k} \bigr)^{\nabla}( t ) \bigr\vert \nabla t \\& \quad \le\alpha^{\frac{k}{q} + ( 1 + p )\frac{k}{p}} \biggl( \int _{0}^{h} \omega^{1 - q} ( t )\Delta t \biggr)^{\frac{k}{q}} \biggl( \int _{0}^{h} \omega ( t ) \bigl\vert f^{\Delta}( t ) \bigr\vert ^{p} \Delta t \biggr)^{\frac{k}{p}} \\& \qquad{}+ ( 1 - \alpha )^{\frac{k}{q} + ( 1 + p )\frac{k}{p}} \biggl( \int _{0}^{h} \omega^{1 - q} ( t )\nabla t \biggr)^{\frac{k}{q}} \biggl( \int _{0}^{h} \omega ( t ) \bigl\vert f^{\nabla}( t ) \bigr\vert ^{p} \nabla t \biggr)^{\frac{k}{p}} \\& \quad \le \biggl( \alpha \int _{0}^{h} \omega^{1 - q} ( t )\Delta t \biggr)^{\frac{k}{q}} \biggl( \alpha \int _{0}^{h} \omega ( t ) \bigl\vert \alpha f^{\Delta}( t ) + ( 1 - \alpha )f^{\nabla}( t ) \bigr\vert ^{p} \Delta t \biggr)^{\frac{k}{p}} \\& \qquad{}+ \biggl( ( 1 - \alpha ) \int _{0}^{h} \omega^{1 - q} ( t )\nabla t \biggr)^{\frac{k}{q}} \\& \qquad{}\cdot \biggl( ( 1 - \alpha ) \int _{0}^{h} \omega ( t ) \bigl\vert \alpha f^{\Delta}( t ) + ( 1 - \alpha )f^{\nabla}( t ) \bigr\vert ^{p} \nabla t \biggr)^{\frac{k}{p}} \\& \quad = \biggl( \alpha \int _{0}^{h} \omega^{1 - q} ( t )\Delta t \biggr)^{\frac{k}{q}} \biggl( \alpha \int _{0}^{h} \omega ( t ) \bigl\vert f^{\diamondsuit _{\alpha}} ( t ) \bigr\vert ^{p} \Delta t \biggr)^{\frac{k}{p}} \\& \qquad{}+ \biggl( ( 1 - \alpha ) \int _{0}^{h} \omega^{1 - q} ( t )\nabla t \biggr)^{\frac{k}{q}} \biggl( ( 1 - \alpha ) \int _{0}^{h} \omega ( t ) \bigl\vert f^{\diamondsuit_{\alpha}} ( t ) \bigr\vert ^{p} \nabla t \biggr)^{\frac{k}{p}} \\& \quad \le \biggl[ \biggl( \alpha \int _{0}^{h} \omega^{1 - q} ( t )\Delta t \biggr)^{k} + \biggl( ( 1 - \alpha ) \int _{0}^{h} \omega^{1 - q} ( t )\nabla t \biggr)^{k} \biggr]^{\frac{1}{q}} \\& \qquad{}\cdot \biggl[ \biggl( \alpha \int _{0}^{h} \omega ( t ) \bigl\vert f^{\diamondsuit_{\alpha}} ( t ) \bigr\vert ^{p} \Delta t \biggr)^{k} + \biggl( ( 1 - \alpha ) \int _{0}^{h} \omega ( t ) \bigl\vert f^{\diamondsuit_{\alpha}} ( t ) \bigr\vert ^{p} \nabla t \biggr)^{k} \biggr]^{\frac{1}{q}} \\& \quad \le \biggl( \alpha \int _{0}^{h} \omega^{1 - q} ( t )\Delta t + ( 1 - \alpha ) \int _{0}^{h} \omega^{1 - q} ( t )\nabla t \biggr)^{\frac{k}{q}} \\& \qquad{}\cdot \biggl( \alpha \int _{0}^{h} \omega ( t ) \bigl\vert f^{\diamondsuit_{\alpha}} ( t ) \bigr\vert ^{p} \Delta t + ( 1 - \alpha ) \int _{0}^{h} \omega ( t ) \bigl\vert f^{\diamondsuit_{\alpha}} ( t ) \bigr\vert ^{p} \nabla t \biggr)^{\frac{k}{p}} \\& \quad = \biggl( \int _{0}^{h} \omega^{1 - q} ( t ) \diamondsuit_{\alpha}t \biggr)^{\frac{k}{q}} \biggl( \int _{0}^{h} \omega ( t ) \bigl\vert f^{\diamondsuit _{\alpha}} ( t ) \bigr\vert ^{p} \diamondsuit_{\alpha}t \biggr)^{\frac{k}{p}} . \end{aligned}$$

 □

### Theorem 2.5

*Assume that* $1 < p \le2$, $q = \frac{p}{p - 1}$, $\alpha \in [ 0,1 ]$, $h \in ( 0,\infty )_{\mathbb{T}}$, $\omega \in\mathbb{C} ( [ 0,h ]_{\mathbb{T}} , ( 0,\infty ) )$ *and* $f \in\mathbb{C}_{\diamondsuit_{\alpha}}^{1} ( [ 0,h ]_{\mathbb{T}} ,\mathbb{R} )$. *If* $\alpha f^{\Delta}\ge 0$, $( 1 - \alpha )f^{\nabla}\ge0$ *and* $f ( 0 ) = 0$, *then*

6 $$ \begin{aligned}[b] &\alpha^{k} \int _{0}^{u} \bigl\vert { \bigl( f^{k} \bigr)^{\Delta}( t )} \bigr\vert \Delta t + ( 1 - \alpha )^{k} \int _{0}^{u} \bigl\vert { \bigl( f^{k} \bigr)^{\nabla}( t )} \bigr\vert \nabla t \\ &\quad\le\sum_{j = 0}^{k - 2} \alpha^{j} \left ( \begin{matrix} k \\ j \end{matrix} \right ) \gamma^{j} \beta ^{\frac{k - j}{q}} \biggl[ \int _{0}^{h} \omega( t ) \bigl\vert g^{\diamondsuit_{\alpha}} ( t ) \bigr\vert ^{p} \diamondsuit_{\alpha}t \biggr] ^{\frac{k - 1}{p}} \\ &\qquad{}+ \alpha^{k - 1} \left ( \begin{matrix} k \\ k - 1 \end{matrix} \right )\gamma^{k - 1} \bigl( f ( h ) - f ( 0 ) \bigr), \end{aligned} $$

*where* $\beta: = \min_{u \in [ 0,h ]_{T} } v ( u )$, $v ( u ) = \max \{ \int _{0}^{u} \omega^{1 - q} ( t )\diamondsuit _{\alpha}t , \int _{u}^{h} \omega^{1 - q} ( t )\diamondsuit_{\alpha}t \} $, $\gamma: = \max \{ \vert f ( 0 ) \vert , \vert f ( h ) \vert \} $.

### Proof

We let $u \in [ 0,h ]_{\mathbb{T}}$ be arbitrary. By applying Theorem 2.4 to the function $g ( t ) = f ( t ) - f ( 0 )$, we obtain

$$\begin{aligned}& \alpha^{k} \int _{0}^{u} \bigl\vert \bigl( f^{k} \bigr)^{\Delta}( t ) \bigr\vert \Delta t + ( 1 - \alpha )^{k} \int _{0}^{u} \bigl\vert \bigl( f^{k} \bigr)^{\nabla}( t ) \bigr\vert \nabla t \\& \quad = \alpha^{k} \int _{0}^{u} \left \vert \sum _{j = 0}^{k - 1} \left ( \begin{matrix} k \\ j \end{matrix} \right ) \bigl( g^{k - j} \bigr)^{\Delta}f^{j} ( 0 ) \right \vert \Delta t \\& \qquad{}+ ( 1 - \alpha )^{k} \int _{0}^{u} \left \vert \sum _{j = 0}^{k - 1} \left ( \begin{matrix} k \\ j \end{matrix} \right ) \bigl( g^{k - j} \bigr)^{\nabla}f^{j} ( 0 ) \right \vert \nabla t \\& \quad \le \left ( \begin{matrix} k \\ 0 \end{matrix} \right ) \biggl[ \alpha^{k} \int _{0}^{u} \bigl\vert g^{k} \bigr\vert ^{\Delta}\Delta t + ( 1 - \alpha )^{k} \int _{0}^{u} \bigl\vert g^{k} \bigr\vert ^{\nabla}\nabla t \biggr] \\& \qquad{}+ \alpha \left ( \begin{matrix} k \\ 1 \end{matrix} \right ) \bigl\vert f ( 0 ) \bigr\vert \biggl[ \alpha^{k - 1} \int _{0}^{u} \bigl\vert g^{k - 1} \bigr\vert ^{\Delta}\Delta t + ( 1 - \alpha )^{k - 1} \int _{0}^{u} \bigl\vert g^{k - 1} \bigr\vert ^{\nabla}\nabla t \biggr] \\& \qquad{}+ \alpha^{2} \left ( \begin{matrix} k \\ 2 \end{matrix} \right ) \bigl\vert f^{2} ( 0 ) \bigr\vert \biggl[ \alpha^{k - 2} \int _{0}^{u} \bigl\vert g^{k - 2} \bigr\vert ^{\Delta}\Delta t + ( 1 - \alpha )^{k - 2} \int _{0}^{u} \bigl\vert g^{k - 2} \bigr\vert ^{\nabla}\nabla t \biggr] \\& \qquad{}\vdots \\& \qquad{}+ \alpha^{k - 2} \left ( \begin{matrix} k \\ k - 2 \end{matrix} \right ) \bigl\vert f^{k - 2} ( 0 ) \bigr\vert \biggl[ \alpha^{2} \int _{0}^{u} \bigl\vert g^{2} \bigr\vert ^{\Delta}\Delta t + ( 1 - \alpha )^{2} \int _{0}^{u} \bigl\vert g^{2} \bigr\vert ^{\nabla}\nabla t \biggr] \\& \qquad{}+ \alpha^{k - 1} \left ( \begin{matrix} k \\ k - 1 \end{matrix} \right ) \bigl\vert f^{k - 1} ( 0 ) \bigr\vert \biggl[ \alpha \int _{0}^{u} \vert f \vert ^{\Delta}\Delta t + ( 1 - \alpha ) \int _{0}^{u} \vert f \vert ^{\nabla}\nabla t \biggr] \\& \quad \le \left ( \begin{matrix} k \\ 0 \end{matrix} \right ) \biggl[ \int _{0}^{u} \omega^{1 - q} ( t ) \diamondsuit_{\alpha}t \biggr]^{\frac{k}{q}} \biggl[ \int _{0}^{u} \omega ( t ) \bigl\vert g^{\diamondsuit_{\alpha}} ( t ) \bigr\vert ^{p} \diamondsuit_{\alpha}t \biggr]^{\frac{k}{p}} \\& \qquad{}+ \alpha \left ( \begin{matrix} k \\ 1 \end{matrix} \right ) \bigl\vert f ( 0 ) \bigr\vert \biggl[ \int _{0}^{u} \omega^{1 - q} ( t ) \diamondsuit_{\alpha}t \biggr]^{\frac{k - 1}{q}} \biggl[ \int _{0}^{u} \omega ( t ) \bigl\vert g^{\diamondsuit_{\alpha}} ( t ) \bigr\vert ^{p} \diamondsuit_{\alpha}t \biggr]^{\frac{k - 1}{p}} \\& \qquad{} + \alpha^{2} \left ( \begin{matrix} k \\ 2 \end{matrix} \right ) \bigl\vert f^{2} ( 0 ) \bigr\vert \biggl[ \int _{0}^{u} \omega^{1 - q} ( t ) \diamondsuit_{\alpha}t \biggr]^{\frac{k - 2}{q}} \biggl[ \int _{0}^{u} \omega ( t ) \bigl\vert g^{\diamondsuit_{\alpha}} ( t ) \bigr\vert ^{p} \diamondsuit_{\alpha}t \biggr]^{\frac{k - 2}{p}} \\& \qquad{}\vdots \\& \qquad{} + \alpha^{k - 2} \left ( \begin{matrix} k \\ k - 2 \end{matrix} \right ) \bigl\vert f^{k - 2} ( 0 ) \bigr\vert \biggl[ \int _{0}^{u} \omega^{1 - q} ( t ) \diamondsuit_{\alpha}t \biggr]^{\frac{2}{q}} \\& \qquad{}\cdot \biggl[ \int _{0}^{u} \omega ( t ) \bigl\vert g^{\diamondsuit_{\alpha}} ( t ) \bigr\vert ^{p} \diamondsuit_{\alpha}t \biggr]^{\frac{2}{p}} \\& \qquad{}+ \alpha^{k - 1} \left ( \begin{matrix} k \\ k - 1 \end{matrix} \right ) \bigl\vert f^{k - 1} ( 0 ) \bigr\vert \alpha \int _{0}^{u} \bigl\vert f^{\Delta}( t ) \bigr\vert \Delta t \\& \qquad{}+ \alpha^{k - 1} \left ( \begin{matrix} k \\ k - 1 \end{matrix} \right ) \bigl\vert f^{k - 1} ( 0 ) \bigr\vert ( 1 - \alpha ) \int _{0}^{u} \bigl\vert f^{\nabla}( t ) \bigr\vert \nabla t \\& \quad \le\sum_{j = 0}^{k - 2} \alpha^{j} \left ( \begin{matrix} k \\ j \end{matrix} \right ) \bigl\vert f^{j} ( 0 ) \bigr\vert \biggl[ \int _{0}^{u} \omega^{1 - q} ( t ) \diamondsuit_{\alpha}t \biggr]^{\frac{k - j}{q}} \biggl[ \int _{0}^{u} \omega ( t ) \bigl\vert g^{\diamondsuit_{\alpha}} ( t ) \bigr\vert ^{p} \diamondsuit_{\alpha}t \biggr]^{\frac{k - j}{p}} \\& \qquad{} + \alpha^{k - 1} \left ( \begin{matrix} k \\ k - 1 \end{matrix} \right )\gamma^{k - 1} \bigl( f ( u ) - f ( 0 ) \bigr). \end{aligned}$$

Similarly,

$$\begin{aligned}& \alpha^{k} \int _{u}^{h} \bigl\vert \bigl( f^{k} \bigr)^{\Delta}( t ) \bigr\vert \Delta t + ( 1 - \alpha )^{k} \int _{u}^{h} \bigl\vert \bigl( f^{k} \bigr)^{\nabla}( t ) \bigr\vert \nabla t \\& \quad \le\sum_{j = 0}^{k - 2} \alpha^{j} \left ( \begin{matrix} k \\ j \end{matrix} \right ) \bigl\vert f^{j} ( 0 ) \bigr\vert \biggl[ \int _{u}^{h} \omega^{1 - q} ( t ) \diamondsuit _{\alpha}t \biggr]^{\frac{k - j}{q}} \biggl[ \int _{u}^{h} \omega ( t ) \bigl\vert g^{\diamondsuit_{\alpha}} ( t ) \bigr\vert ^{p} \diamondsuit_{\alpha}t \biggr]^{\frac{k - j}{p}} \\& \qquad{}+ \alpha^{k - 1} \left ( \begin{matrix} k \\ k - 1 \end{matrix} \right )\gamma^{k - 1} \bigl( f ( h ) - f ( u ) \bigr). \end{aligned}$$

Adding these two inequalities and taking into account that $a^{r} + b^{r} \le ( a + b )^{r}$ holds, for $a,b \ge0$ and $r \ge 1$, yield the desired inequality. □

## Conclusion

In this paper, we have obtained several Opial-type integral inequalities on time scales via the notion of the diamond-alpha derivative. These inequalities are related to the function $f^{k}$.
